# A Study of the Regulatory Mechanism of the CB1/PPARγ2/PLIN1/HSL Pathway for Fat Metabolism in Cattle

**DOI:** 10.3389/fgene.2021.631187

**Published:** 2021-05-04

**Authors:** Ruili Liu, Xianxun Liu, Xuejin Bai, Chaozhu Xiao, Yajuan Dong

**Affiliations:** ^1^Laboratory of Animal Physiology and Biochemistry, Animal Embryo Center, College of Animal Science, Qingdao Agricultural University, Qingdao, China; ^2^Laboratory of Animal Molecular Shandong Black Cattle Breeding Engineering Technology Center, College of Animal Science, Qingdao Agricultural University, Qingdao, China

**Keywords:** CB1, HSL, fat metabolism, cattle, pathway

## Abstract

Fat metabolism is closely related to the economic characteristics of beef cattle. Therefore, regulating fat deposition and increasing intramuscular fat deposition are among the main goals of breeders. In this study, we aim to explore the regulatory role of CB1 gene on PPARγ2/PLIN1/HSL pathway in fat metabolism, and to further explore the differential expression of regulatory factors of this pathway in Shandong black cattle and Luxi cattle. In this study, CB1 overexpression stimulated lipid synthesis in adipocytes to some extent by increasing the levels of FASN and ACSL1. CB1 inhibitors reduce the lipid content in adipocytes and reduce the expression of GLUT1 and Insig1. In addition, overexpression of CB1 decreased the expression of PPARγ2 and led to an increase in PLIN1 expression and a decrease in HSL expression in adipocytes. We also found that the CB1/PPARγ2/PLIN1/HSL was differentially expressed in the different breeds of cattle and was involved in the regulation of fat metabolism, which affected the fatty acid content in the longissimus dorsi muscle of the two breeds. In short, CB1 participates in lipid metabolism by regulating HSL in the PPARγ2 and PLIN1 pathways, and improves lipid formation in adipocytes. In conclusion, CB1/PPARγ2/PLIN1/HSL pathway may be involved in the regulation of lipid metabolism.

## Introduction

Fat metabolism is closely related to the economic characteristics of beef cattle. The deposition of animal body fat represents a balanced state of fat synthesis and catabolism. Once the original balance is destroyed, fat deposition is increased or body fat is reduced, thus affecting the meat quality of animals. Therefore, regulating fat deposition and increasing intramuscular fat deposition are among the main goals of breeders. Currently, increasing research is focused on the mechanism of animal fat deposition and its regulation to influence the processes of animal fat deposition, improve carcass quality and increase production levels.

The endogenous cannabinoid system, a ubiquitous lipid signaling system, plays an important regulatory role in the energy metabolism of all vertebrates. Cannabinoid receptor 1 (CB1) was first discovered by Devane et al. (1988). Matsuda and others found that CB1 was mainly expressed in the nervous system and peripheral tissues (Matsuda et al., 1990). The results showed that the endogenous cannabinoid system of the central nervous system can affect the feeding center to regulate appetite, and its antagonists can effectively inhibit appetite (Zou and Kumar, 2018). The transcription abundance of CB1 was significantly higher in highly fertile bulls than in bulls with low fertility, indicating that it is significantly related to the fertility of bulls (Osei-Hyiaman et al., 2005; Kumar et al., 2018). Previous studies have also shown that CB1 can activate multiple intracellular signaling pathways, which can negatively regulate CPT1 through the PPARα signaling pathway, thus affecting the decomposition of fat (Wei et al., 2018). The addition of the Δ-9-THC activator of CB1 to pig adipocytes *in vitro* resulted in a decrease in PPARα and CPT1 expression and an increase in lipid droplet deposition in adipocytes (Wei et al., 2013). On the contrary, the addition of CB1 inhibitor SR141716A will increase the expression of PPARα and carnitine palmitoyltransferase (CPT1), and will reduce intracellular fat deposition (Wei et al., 2013). Furthermore, CB1 could inhibit lipolysis by inhibiting hormone-sensitive lipase (HSL) activity, but it could also inhibit CPT2 activity, reducing the fatty acids entering mitochondria and thus negatively regulating lipolysis, although the specific mechanism needs to be studied (Shen and Wang, 1997).

Evidence shows that cannabinoid receptor 1 (CB1) is involved in fatty acid metabolism and the regulation of energy metabolism. However, HSL is an important lipolytic enzyme involved in PPARγ2/Perilipin1(PLIN1)/HSL pathway. Transcriptome data processing predicts that CB1 can affect HSL gene expression, but its regulatory mechanism is still unclear. In order to further explore the regulatory effect of CB1 on PPARγ2/PLIN1/HSL pathway gene in fat metabolism, a CB1 overexpression vector was constructed and overexpressed in 3T3-L1 adipocytes by liposome transfection. Meanwhile, the CB1 gene was inhibited by inhibitor SR141716A. Objective to observe the effect of CB1 on PPARγ2/PLIN1/HSL pathway and the expression of lipid metabolism related proteins, and to explore the regulatory mechanism of CB1 on PPARγ2/PLIN1/HSL pathway. Furthermore, PPARγ2/PLIN1/HSL pathway was differentially expressed in Shandong black cattle and Luxi cattle. At the level of gene expression, the molecular mechanism by which fat metabolism is regulated in cattle was explored, and important candidate genes affecting fat metabolism were found, which provided new prospects for improving meat quality and breeding to generate new breeds.

Shandong black cattle were the first domestic bovine breed obtained by the transfer of vitrified frozen somatic cell-cloned embryos. They have been carefully bred by researchers, and the bulls are used for breeding. Researchers have overcome the shortcomings of Luxi cows (female parent) by crossbreeding and molecular marker-assisted breeding. They have improved Luxi cattle by using semen from Shandong black-haired bulls (male parent) and mixed families, such that the offspring exhibit a combination of excellent characteristics and have improved production performance. In 2015, the breed was recognized as a new population by experts and was established as a Chinese-type germplasm for breeding new varieties. Luxi cattle are among the five local beef cattle breeds in China. They have high meat production capacity, tender meat with long-lasting freshness, and a reputation of having “five flavors and three layers of meat” (Lv, 2015). With the approval of the local government, we have established the local standard of Shandong black cattle. The major genes FABP4, MSTN and HSL related to fat deposition, muscle tenderness and marbling have been preliminarily screened (Liu et al., 2020). As a molecular marker for early screening of cattle, ultrasonic was used to determine marbling, eye muscle area, back fat thickness and intramuscular fat content. The purpose of this study was to investigate the molecular mechanism by which fat metabolism is regulated in cattle, and important candidate genes affecting fat metabolism were found in Hybrid Progenies (Shandong black cattle) and primordial maternal generation (Luxi cattle), and further explore its regulation related to production performance. Finally, high-quality beef cattle were selected to realize the leap from new varieties to new varieties.

## Materials and Methods

### Construction of a Regulatory Network of Fat Metabolism

The construction of a regulatory network of fat metabolism was based mainly on two types of data: the data from the transcriptome database and research results established in our laboratory and the data obtained from information related to fat metabolism in literature databases such as NCBI, CNKI and Ensembl. We have stored the RNA sequence data in the public domain GEO NCBI to obtain the GEO accession numbers: GSM4904154, GSM4904155, GSM4904156, GSM4904157, GSM4904158, GSM4904159. Based on the existing data and information, a crude model of fat decomposition was constructed, and a regulatory pathway of fat mobilization was added. Based on transcriptome data, we have obtained 1,413 differential genes. To understand the functions of DEGs (Different Expression Genes), goatools^1^ and KOBAS^2^ were used to conduct analyses of GO functional enrichment and Kyoto Encyclopedia of Genes and Genes (KEGG) pathway enrichment. When the *q*-value of a DEG was less than 0.05, the GO and KEGG pathways were considered to be significantly enriched. The GO enrichment analysis for DGEs was performed using clusterProfiler v3.16.0 in the R package. The PPI network of DEGs was analyzed using the STRING^3^ database, which contains direct and indirect protein associations. Some high-frequency but unclear regulatory genes related to fat decomposition were found. We analyzed the metabolic pathways identified in the KEGG database and the complete transcriptome database established in our laboratory to speculate the possible regulatory mechanisms (Liu et al., 2020). In this experiment, CB1 was selected to regulate HSL, which was an approach that had not been previously explored. The experiment was used to analyze and verify the collected data used to improve the constructed regulatory network of fat metabolism.

### Tissue Sample Collection

The survey was conducted in strict accordance with the recommendations of the National Institutes of Health Guide for the care and use of laboratory animals. The scheme was approved by IACUC (Institutional Animal Care and Use Committee) (Zhao et al., 2004). The animals used in the experiment, Shandong black cattle (male), Luxi cattle (male) were obtained from Shandong Black Cattle Technology Co., Ltd. Healthy 18-months-old Shandong black cattle and Luxi cattle were used, and three cattle in each group were selected. After slaughtering, 2–5 g of heart, liver, spleen, lung, kidney, longissimus dorsi muscle and fat were collected immediately and stored in liquid nitrogen. The tissue samples were stored in 4% paraformaldehyde.

### Cell Culture and Transfection

The pcDNA3.1(+) plasmid and the CB1 inhibitor SR141716A were purchased from Qingdao Saishang Biotechnology Co., Ltd., for cell transfection according to the manufacturer’s instructions (Transfection kit: C0511). The CB1 fragment and pcDNA3.1(+) plasmid were digested by restriction endonucleases *Eco*RI and *Bam*HI, and eukaryotic plasmid vectors were constructed according to the instructions of the T4DNA ligase kit (Cat No.: D7006) and plasmid extraction kit (Cat No.: D0020). The 3T3-L1 preadipocyte line was purchased from cell bank of Chinese Academy of Sciences. DMEM medium (GIBCO, Cat No.: SH30022.1, NaHCO3 1.5 g/L added) was purchased from Beijing Soleibao Co., Ltd. Fetal bovine serum (Cat No.: 11011-8611, 20%; double antibody: 1%) was purchased from Zhejiang Tianhang Biotechnology Co., Ltd. The cells were cultured in a dish and divided into four groups: a negative control group (NC-OE), overexpression group (CB1-OE), negative control group (NC-inhibitor) and inhibitor (CB1-inhibitor). A total of 75–80% of the fusion cells were transfected by the liposome transfection technique. The CB1 inhibitor was added at a concentration of 0.025 pmol/well. The cells were collected and analyzed 48 h after transfection (Lian et al., 2016).

### qRT-PCR

One milliliter of TRIzol reagent (Roche) was added to homogenate the cells, and the total RNA was extracted. RNA was reverse transcribed into cDNA, and cDNA was used as a template for fluorescence quantitative PCR. The 20-μL PCR system included 1 μL of template cDNA, 10 μL of iTaq Universal SYBR Green Supermix, 0.6 μL of upstream primer, 0.6 μL of downstream primer (primers are shown in Supplementary Table 2), and 7.8 μL of RNA-free water. The following PCR procedure was used: pre-denaturation at 94°C for 10 min; 40 cycles of denaturation at 94°C for 30 s, annealing at 60°C for 30 s, elongation at 72°C for 40 s. Each sample was subjected to PCR three times. Glyceraldehyde-3-phosphate dehydrogenase (GAPDH) was used to standardize gene expression data. The Ct value of each sample was measured and the average value was calculated. Finally, The expression of the target gene in each sample was calculated by the 2^–ΔΔ*CT*^ value method.

### Western Blot Analysis

Cell and tissue samples were lysed in RIPA buffer containing a protease inhibitor cocktail. The BCA kit was purchased from Shanghai Beiyang Times Biotechnology Research Institute, China, and was used to determine protein concentration (Wang et al., 2016). Supplementary Table 2 is the main primary antibody (ABS) related information. The extracted protein was concentrated and separated by SDS-PAGE and then transferred to a PVDF membrane for WB analysis. According to the size of maker, PVDF membrane was cut into small pieces for incorporation to incubate antibody (as if a standard practice). The primary antibodies were ABS (1:500) and GAPDH (1:1,000), and the secondary antibody was horseradish peroxidase-labeled goat anti-rabbit IgG (H + L) (1:2,000). The specific steps were followed according to the literature (Jin and Kennedy, 2015). A luminescence kit was used to expose the proteins, which were developed on negative film in a darkroom and scanned. Specifically, ECL light-emitting substrate exposure was captured and developed on film that was scanned and analyzed with ImageJ 1.39u software to determine the optical density value of the target strip. The band strength of each specific protein was normalized to that of GAPDH and then to the NC/control. The growth-related proteins Smad2, Pi3K, TAK1, and IGF1R and the lipid metabolizing enzymes FASN, ACSL1, GLUT1, and Insig1 were analyzed.

### Immunohistochemistry (IHC)

The harvested cells were fixed in 4% paraformaldehyde for 1 h, then laid on a microscope slide coated with poly-lysine and air-dried. After being washed 3 times with PBS, the cells were incubated in PBS for 1 h at RT. The cells were blocked in PBS with 1% (wt/V) BSA and 1% goat serum for 30 min and then incubated overnight at 4°C with the primary antibody diluted with blocking solution (Table 1).

**TABLE 1:** Primary antibody information.

**Gene**	**Cat.#**	**Size**
GAPDH	bs-13282R	45 kda
CB1	bs-1683R	52 kda
PPARγ2	bs-4888R	57 kda
PLIN1	bs-10779R	57 kda
HSL	bs-0455R	116 kda
Smad2	bs-0718R	52 kda
Pi3K	bs-5571R	80 kda
TAK1	bs-3585R	67 kda
IGF1R	bs-0227R	78 kda
FASN	bs-1498R	272 kda
ACSL1	bs-5022R	77 kda
GLUT1	bs-0472R	54 kda
Insig1	bs-5074R	30 kda

Tissue sample slices (5 mm) were prepared and subjected to antigen retrieval and immunostaining as previously described (Liu et al., 2019). Briefly, anti-virus antibody (1:150) was added (Table 1) to a tissue sample slice and put in a refrigerator at 4°C overnight. The second antibody (Anti-Rabbit IgG (whole molecule): A0545) was added to cover the tissue slice, which was place in a 37°C incubator for 30 min. Wash the sections 3 times with PBS buffer. DAPI dye was dripped onto each section at room temperature and allowed to stand for 5 min. The film was sealed with an anti-fluorescence attenuator and protected from light. Fluorescence images were obtained using a Leica laser scanning confocal microscope (LEICA TCS SP5 II, Germany) (Liu et al., 2016). The number of positive cells was counted and normalized to that of the NC or the virgin-stage cells. Duplicate experiments were repeated 4–6 times.

### Fatty Acid and Flavor Substance Determination

In the experiment, we selected 10 Shandong Black Cattle and Luxi Cattle each and took their longissimus dorsi. The determination of fatty acids refers to the fatty acid hydrolysis method <GB 5009.168-2016> specified in the national food safety standards. 7890A-7000 gas chromatography-mass spectrometer and 6890 gas chromatograph used in the test were purchased from Agilent Technology Co., Ltd. and db-wax chromatographic column (30 m × 250 μm, 0.25 μm) from J & W company. Animal fatty acid methyl esters were prepared according to <GB/T 9695.2-2008>. Firstly, in the reflux reaction, 12–15% boron trifluoride methanol solution was added, saturated sodium chloride solution and isooctane were added to obtain the appropriate sodium hydroxide methanol solution. Then the solutions are mixed and extracted to obtain an extract, which is analyzed by gas chromatography with an isooctane layer. The method of determination of flavor substances is to transfer the extract to GC-MS, desorb at 250°C for 7 min, then start the instrument to collect two sets of data, and then enter it into the Excel2007 table. According to the calculation formula of <gb9695.2-2008>, the data were processed.

### Statistical Analysis

Statistical analysis was performed using SPSS 19.0 (SPSS, Michigan Avenue, Chicago, Illinois, United States). All data were expressed as means ± standard errors of means (SEM). Prior to statistical analysis, the normality of the data was verified using the Kolmogorove-Smirnov test, and the homogeneity of variances was analyzed by Levene’s test. When the data satisfied the assumptions of homogeneity of variances, the differences among the treatments were evaluated by one-way ANOVA and Tukey’s multiple range test. The data comparisons between two groups were performed using Student’s *T*-test for independent samples. Difference was considered significant at *P* < 0.05.

## Results

### Construction of the Regulatory Network of Fat Metabolism

**FIGURE 1:**
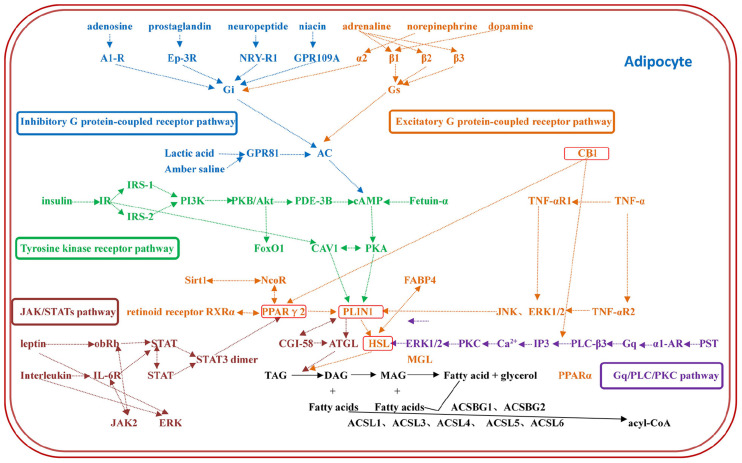
The regulatory network of fat metabolism in adipocytes. The regulatory network of fat metabolism includes five complete regulatory pathways: the excitatory G protein-coupled receptor pathway, inhibitory G protein-coupled receptor pathway, tyrosine kinase receptor pathway, Gq/PLC/PKC pathway and JAK/STAT pathway. Each color represents a pathway, and the most regulated genes are PLIN1 and HSL, which are the terminal regulators of multiple integrated pathways.

The constructed regulatory network of fat metabolism was based on four parts: mobilization of fat, decomposition of glycerol, β-oxidation of mitochondrial fatty acids and β-oxidation of peroxygenase fatty acids. It was found that the catalytic enzymes in each step of the four parts play important roles in fat metabolism. In addition, the genes that play a role in fat metabolism included TNF-α, CB1, Sirt1, FABP4, AQP7, AQP9, FoxO1, CGI-58, and fetuin-α. The whole regulatory pathway mainly mobilizes fat and included the excitatory G protein-coupled receptor pathway, inhibitory G protein-coupled receptor pathway, tyrosine kinase receptor pathway, Gq/PLC/PKC pathway and JAK/STAT pathway (Figure 1). The most regulated genes were PLIN1 and HSL, which were the terminal regulatory points for multiple integrated pathways (Table 2). Combined with the results from screening of the bovine transcriptome database in the laboratory, signaling pathways associated with focal adhesion, regulation of lipolysis in adipocytes, and ECM-receptor interactions and the AMPK signaling pathway, PPAR signaling pathway and adipocytokine signaling pathway were found to be the enriched typical pathways, and the number of DEGs regulating lipolysis in adipocytes was 13. The results from the analysis showed that FABP4, PCK1, ADIPOQ, HSL, SCD5, PIK3R3 and PLIN1 were enriched in five pathways (attachment 1: Supplementary Table 1, a list of significantly enriched KEGG pathways related to bovine lipid metabolism).

**TABLE 2:** Signal transduction mechanisms of fat metabolism.

**Pathway**	**Hormone**	**Membrane receptor**	**G-protein**	**Effector protein**	**Protein kinase**	**Functional protein**
Excitatory G protein-coupled receptor pathway	Adrenaline	β1	Gs	AC	PKA	PLIN1
	Dopamine	B2				HSL
		B3				
Inhibitory G protein-coupled receptor pathway	Noradrenaline Prostaglandin	α2	Gi	AC	PKA	PLIN1
	Neuropeptide	Ep-3R				HSL
		NPY-R				
Gq/PLC/PKC pathway	PST	α1-AR	Gq	PLC-β	PKC	ERK1/2
						HSL

### CB1 Affects Factors Related to Cell Growth

**FIGURE 2:**
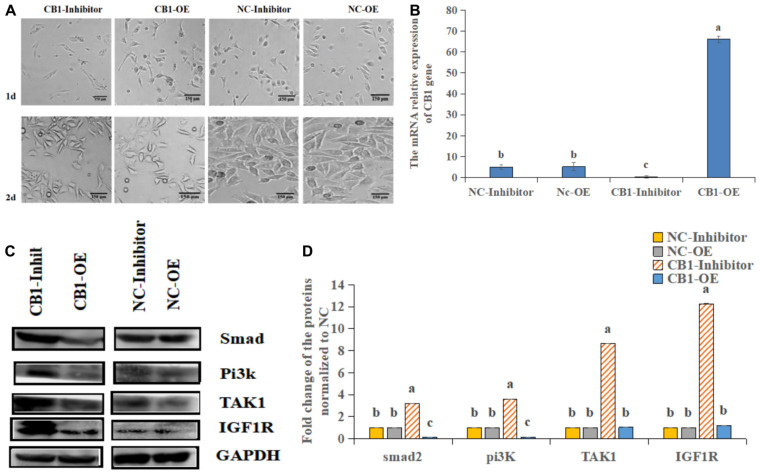
The effect of a CB1 overexpression or inhibitor on the growth of preadipocytes (3T3-L1). **(A)** Preadipocytes growth diagram of CB1 simulant or inhibitor treatment for 48 h. **(B)** CB1 expression was measured 24 h after initiation of CB1 overexpression or application of the inhibitor treatment, and the quantitative data showed multiple changes compared to the NC. **(C)** Western blotting was used to detect CB1 overexpression- or inhibitor-induced changes in cell growth-related protein levels. In the experiment, according to the size of maker, we cut PVDF membrane and incubate different protein antibodies, respectively. In the results, the bands are presented independently. **(D)** The quantitative data of the Western blotting are presented as fold changes compared to the NC data after ImageJ quantification; a, b, c indicate significant differences among the different treatments (*n* = 3; *P* < 0.05).

First, we analyzed the expression of CB1 after transfection (Figure 2A). CB1 expression in the transfected cells was detected by real-time RT-PCR. It was found that overexpressing CB1 increased CB1 expression by nearly 12-fold compared to transfecting with NC-OE, and the CB1 inhibitor decreased CB1 expression by approximately 29-fold compared to that induced by the NC inhibitor (Figure 2B), indicating that CB1 overexpression and the CB1 inhibitor had considerable effects.

To study the function of CB1, we studied the effect of CB1 expression on cell growth and proliferation. Forty-eight hours after transfection, the factors related to cell growth, namely, Pi3K, IGF1R, ATK1, and Smad2, were measured. CB1 overexpression did not alter TAK1 or IGF1R expression; however, their expression was increased by the CB1 inhibitor. CB1 overexpression decreased Smad2 and Pi3K expression, but the expression of these proteins was increased by the CB1 inhibitor (Figures 2C,D). The data indicate that CB1 may have some effect on the growth of preadipocytes.

### CB1 Participates in Fat Metabolism

**FIGURE 3:**
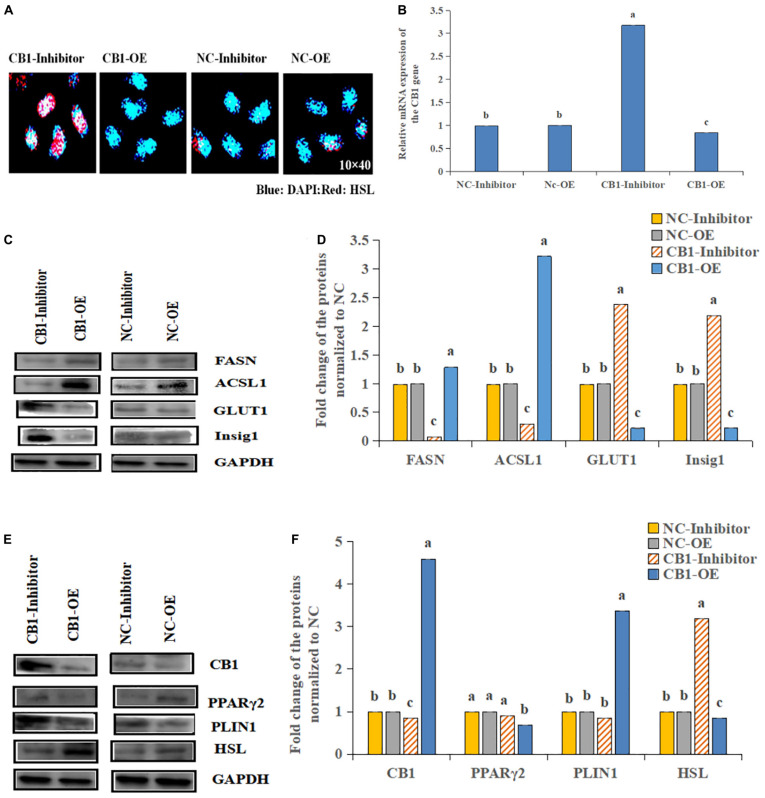
Effects of CB1 overexpression or inhibition on the lipid metabolism of adipocytes. **(A)** CB1 overexpression decreased and the CB1 inhibitor increased the levels of the HSL protein, as shown by IHC. Blue represents nucleus, Red represents HSL positive expression. **(B–F)** are the same as Figure 2.

The HSL expression was increased by the CB1 inhibitor but decreased by CB1 overexpression (Figure 3A). Because CB1 expression changed the cell content of hormone-sensitive esterase, the enzymes related to lipid metabolism were determined. The levels of FASN and ACSL1 were increased by the CB1 overexpression but decreased by the CB1 inhibitor (Figure 3B). Other lipid-metabolizing enzymes, Insig1 and GLUT1, increased under the action of the inhibitors but decreased when CB1 was overexpressed (Figures 3C,D). These data suggest that CB1 may participate in cell lipid metabolism by changing the levels of lipid-metabolizing enzymes. In addition, CB1 may change the protein levels of fatty acid synthetase (FASN) and glucose transport (GLUT1). 48 h after transfection, CB1 overexpression decreased PPARγ2 and HSL expression, which was significantly increased by the CB1 inhibitor. PLIN1 expression was significantly increased by CB1 overexpression but was decreased by the CB1 inhibitor (Figures 3E,F).

### Expression Differences in the CB1/PPARγ2/PLIN1/HSL Pathway Among the Different Breeds of Cattle

**FIGURE 4:**
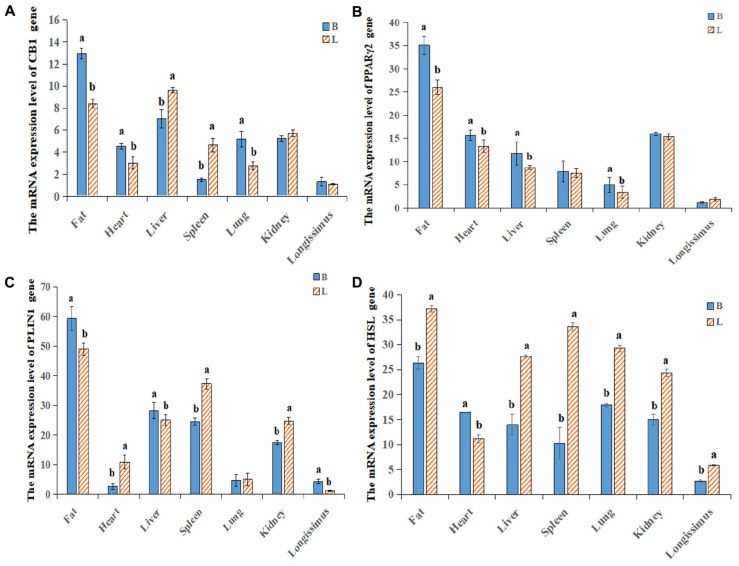
Relative expression levels of the CB1/PPARγ2/PLIN1/HSL genes in different tissues. **(A–D)** qRT-PCR was used to detect the expression levels of CB1, PPARγ2, PLIN1 and HSL in different tissues. There was a difference in the expression between Shandong black cattle and Luxi cattle; B represents Shandong black cattle; L represents Luxi cattle; a and b were significantly different because of the different treatments (*n* = 3; *P* < 0.05).

**FIGURE 5:**
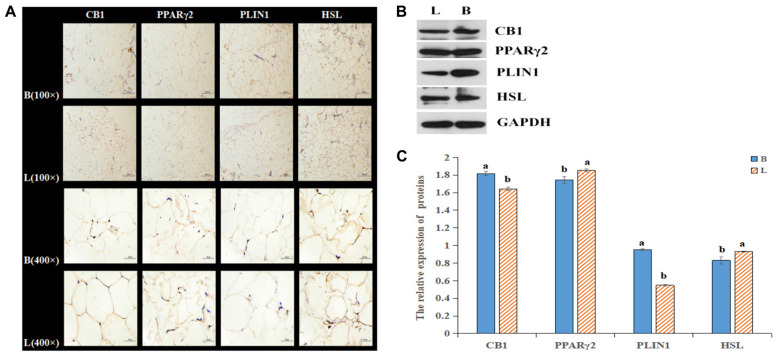
Relative expression of the four proteins in tissues. **(A)** Relative expression of the four proteins in tissues as shown by IHC. **(B)** The protein levels of CB1, PPARγ2, PLIN1 and HSL were detected by Western blotting. **(C)** The quantitative data from the Western blot analyses, as determined by Image J, indicated that the protein levels were changed, and the significant differences were the same as those described above.

By analyzing the expression profiles of CB1, PPARγ2, PLIN1, and HSL in different tissues of the cattle (Figures 4A–D), it was found that the expression levels of the four genes were the highest in fat, followed by the levels in the liver, spleen and kidney. Because the longissimus dorsi muscle contains intramuscular fat, there was a low level expression, exerting a certain effect on intramuscular fat. Furthermore, it was found that CB1, PPARγ2, PLIN1, and HSL were expressed differentially between the two breeds of cattle. It was found that CB1 and HSL were expressed in the cell membrane and that PPARγ2 and PLIN1 were expressed in the cytoplasm (Figure 5A). Western blot analysis showed that the expression levels of CB1, PPARγ2, PLIN1 and HSL protein were different, among which CB1 and PPARγ2 were relatively high (Figures 5B,C). In addition, there were significant differences in the expression of the four proteins between the two breeds of cattle, and the expression had different effects on fat metabolism in the different breeds of cattle.

### Determination of the Fatty Acids in Beef

**FIGURE 6:**
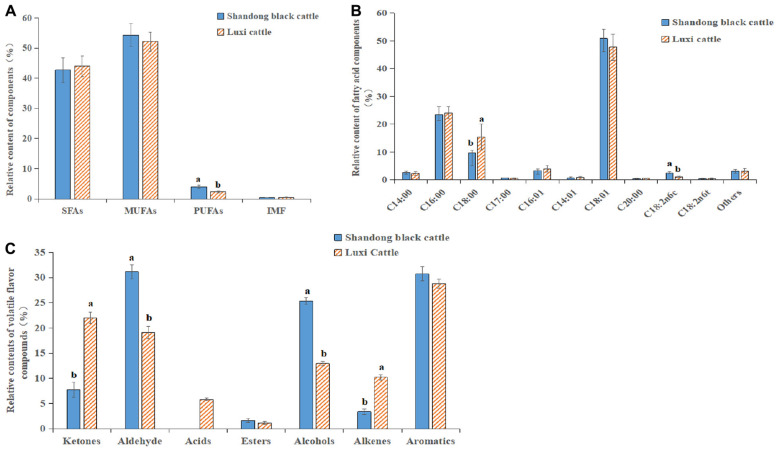
Fatty acid and Flavor substance determination. **(A)** SFAs, saturated fatty acids; MUFAs, monounsaturated fatty acids; PUFAs, polyunsaturated fatty acids; IMF, Intramuscular fat content. **(B)** Relative contents of various fatty acid classes in Longissimus dorsi between Shandong black cattle and Luxi cattle, detailed information in Abbreviations. **(C)** Relative contents of volatile flavor compounds in Longissimus dorsi between Shandong black cattle and Luxi cattle. The significant differences were the same as those described above.

The content of fatty acids in the beef of the Shandong black cattle (B) and Luxi cattle (L) groups was determined, as shown in Figure 6A. In the muscle tissues of the two kinds of cattle, the content of saturated fatty acids (SFAs) was low, and the content of monounsaturated fatty acids (MUFAs) was high; the content of polyunsaturated fatty acids (PUFAs) in the Shandong black cattle was significantly higher than it was in the Luxi cattle. Among these fatty acids, the content of stearic acid (C18:0) (saturated fatty acid) in Shandong black cattle is significantly lower than that of Luxi cattle, while the content of linolenic acid (C18: 2n6c) (polyunsaturated fatty acid) is significant higher than Luxi cattle (Figure 6B). The ratio of unsaturated fatty acids to saturated fatty acids in the muscle tissue of the Shandong black cattle and Luxi cattle was 1.73:1 and 1.34:1, respectively. There were significant differences in the composition of volatile flavor compounds between the two groups (Figure 6C). The highest relative content of Shandong black cattle group was aldehydes (31.13%), followed by aromatics (30.71%) and alcohols (25.35%); the highest relative content of Luxi Cattle group was aromatics (28.76%), followed by ketones (22.02%) and aldehydes (19.07%).

## Discussion

In this study, the construction of the regulatory network of fat metabolism was based mainly on two types of data: the data from the transcriptome database and the research results established in our laboratory and the data obtained from information related to fat metabolism in literature databases such as NCBI, CNKI and Ensembl. In addition, six pathways related to fat growth and development were found in the existing transcriptome database. Further analysis of pathway enrichment showed that PPAR signaling was upregulated significantly, and some metabolism-related pathways were regulated differentially (Khan et al., 2019, 2020a; Chen et al., 2020). Among them, HSL and PLIN1 were significantly enriched during the regulation of lipolysis in adipocytes and in the AMPK signaling pathway, the adipocytokine signaling pathway and the PPAR signaling pathway, which are involved in the regulation of multiple fat metabolism pathways; therefore, HSL and plin1 were selected as important candidate marker genes for beef fat decomposition (Junjvlieke et al., 2020; Khan et al., 2020b). Combined with the gene network of fat metabolism constructed in this study, there are five fat metabolism pathways: the excitatory G protein-coupled receptor pathway, inhibitory G protein-coupled receptor pathway, Gq/PLC/PKC pathway, tyrosine kinase receptor pathway and JAK/STAT pathway. The first three pathways are in the G protein-coupled pathway. After G-protein activation, the activity of effector proteins is initiated or inhibited, thus leading to activated protein kinase and changes in the activity of functional proteins that either accelerate or impede the process of fat decomposition (O’Hayre et al., 2014; Pathak et al., 2017). In this study, the most regulated genes were PLIN1 and HSL, which are terminal regulators of multiple integrated pathways and play important roles in lipolysis. PPARγ is an important nuclear transcription factor in adipocytes. Its expression product in adipocytes is PPARγ2. Because of a promising element, ppre, in the promoter region of PLIN1, the expression of PLIN1 is directly regulated by PPARγ (Arimura et al., 2004). CB1 has been shown to regulate PPAR gene expression and further regulate fat metabolism (Cota et al., 2003). In addition to the complete regulatory pathway, the genes promoting lipolysis include TNF-α, Sirt1, FABP4 and AQP7, and the genes inhibiting lipolysis include CB1 and fetuin-α. Wei et al. (2018) and other researchers found that the use of inhibitors to reduce the expression of CB1 can significantly increase the expression of HSL, indicating that CB1 reduction can increase the expression of key genes involved in fat decomposition and thus promote fat decomposition, but the specific regulatory mechanism is not clear. On the basis of this study, we speculated that CB1 regulation of HSL might be mediated by PPARγ2 and PLIN1, which may be involved in the regulation of bovine fat metabolism.

In this study, CB1 overexpression did not alter TAK1 and IGF1R; however, these proteins were increased by the CB1 inhibitor. CB1 overexpression decreased Smad2 and Pi3K expression, which was increased by the CB1 inhibitor. Referring to the literatures (Galvan et al., 2003; Onodera et al., 2019), we found that the decrease of Smad2 and PI3K expression can inhibit cell proliferation, and the increase of TAK1 and IGF1R expression can promote cell growth and proliferation. In order to prove the proliferation of adipocytes. The above results indicate that overexpression of CB1 can inhibit the proliferation of adipocytes to a certain extent. CB1 inhibitor can promote the proliferation of adipocytes. In conclusion, CB1 may have some effect on the growth of preadipocytes. In addition, we found that CB1 expression changed the FASN, ACSL1 and Insig1 genes, which were related to lipid metabolism, and thus regulated 3T3-L1 lipid metabolism *in vitro*. Previous studies have shown that INSIG1 can reduce the synthesis of fatty acids and triglycerides, and inhibit the lipid metabolism of adipocytes (Luo et al., 2017). Inhibition of CB1 inhibited lipid formation, leading to a decrease in the level of Insig1 protein. CB1 overexpression could promote the increase in FASN and ACSL1 protein levels. FASN and ACSL1 promoted the intake of fatty acids and the synthesis of triglycerides, further promoted the lipid metabolism of adipocytes, and finally increased the lipid content. Furthermore, CB1 overexpression reduced the level of glucose transporter 1 (GLUT1) protein; however, the CB1 inhibitor increased it. These data indicate that CB1 changes not only the levels of lipid formation in 3T3-L1 but also the effect of glucose uptake, and CB1 may participate in glucose transport.

In this study, CB1 overexpression decreased PPARγ2 and HSL expression, which was significantly increased by the CB1 inhibitor. PLIN1 expression was significantly increased by CB1 overexpression but was decreased by the CB1 inhibitor. It is suggested that CB1 is negatively correlated with PPARγ2 and HSL in bovine preadipocytes and positively correlated with PLIN1. In the past, CB1 agonists were used to treat intramuscular fat cells. With increasing CB1 agonist concentration, the mRNA level of the HSL gene decreased; with increasing amounts of the CB1 inhibitor SR141716AA, the mRNA level of HSL increased (Wei et al., 2018). This is consistent with the results of this study. Then, there was a significant positive correlation between CB1 and HSL in the liver of pigs, and no significant correlation between CB1 and HSL in the peritoneal omentum, the longissimus dorsi, perirenal fat, and subcutaneous fat (Wei, 2014). This finding is not consistent with the results of CB1-mediated negative regulation of HSL described in this paper. It is possible that the simulated survival conditions are quite different from the actual situation of the animal as a whole and cannot fully represent the physiological functions of the animal body under general conditions. In this study, the expression profiles of the four genes showed that they all expressed the highest degree in fat, indicating that they play an important role in the development and growth of adipose tissue. Further research found that the expression trends of the four proteins showed regularity, and the expression of CB1 showed the opposite trend with HSL, and showed the same trend with PPARγ2 and PLIN1. It is interesting to find that the results of tissue level and cell level are basically the same. At the same time, we will further study the correlation between the expression of four proteins at the tissue level. However, in intramuscular fat, CB1 expression was negatively correlated with PPARγ2 and HSL and positively correlated with PLIN1, as was the regulation of the protein level. These differences may be due to the complex mechanism of CB1 regulation of HSL *in vivo* and the simultaneous effects of multiple pathways, and it can be shown that CB1 negatively regulates HSL through the PPARγ2 and PLIN1 genes.

The expression of CB1 and PLIN1 in the Shandong black cattle was significantly higher than it was in the Luxi cattle, but the PPARγ2 and HSL expression levels were significantly lower than they were in the Luxi cattle. Previous studies have shown that the rate-limiting enzyme of triacylglycerol (tag) catabolism in hormone-sensitive lipase (HSL) tissue, together with triacylglycerol lipase and monoacylglycerol lipase, regulates the storage of fat in muscle (Liu et al., 2015). PPARγ2 plays an important role in regulating lipid metabolism, adipogenesis, insulin sensitivity, inflammatory response, cell growth and differentiation. Among these important roles, the role of PPARγ2 with known target genes (PLIN1, PLIN3, HSL, and FABP4) is related to almost all aspects of fat metabolism and affects the transcription of various fatty acid transport and metabolism genes (Huang et al., 2007; Yang et al., 2017; Liu et al., 2018). In the Shandong black cattle and Luxi cattle, the results showed that the ratio of unsaturated fatty acids to saturated fatty acids was 1.73:1 and 1.34:1, respectively. Beef flavor is directly related to fatty acids. Compared with SFA, the composition of flavor compounds is easily influenced by the oxidation products of PUFA (Insausti et al., 2005). In this study, we found that aldehydes have a very low aroma threshold, which can give pleasant sweet and fruit flavor to beef, so they are very important characteristic aroma components of beef (Keys et al., 1965). The relative content of aldehydes in longissimus dorsi muscle of Luxi cattle was significantly lower than that of Shandong black cattle (*P* < 0.05). Acids can bring unpleasant smells to beef, such as acid and urine. Only the acids detected in the fattening group may affect the flavor of yak meat in the fattening group. These data indicated that the ratio of beef fatty acids was more suitable and that the flavor was better in the Shandong black cattle than they were in the Luxi cattle.

In conclusion, CB1 changes the lipid metabolism of preadipocytes *in vitro*. CB1 overexpression can promote the increase expression of FASN and ACSL1 protein, indicating that CB1 was involved in lipid metabolism. In addition, CB1 participated in lipid metabolism through PPARγ2 and PLIN1 mediated regulation of HSL, and improves lipid formation in adipocytes. In conclusion, CB1/PPARγ2/PLIN1/HSL pathway may be involved in the regulation of lipid metabolism.

## Data Availability Statement

The datasets used and/or analyzed in the current study are available from the corresponding author upon reasonable request.

## Ethics Statement

The animal study was reviewed and approved by the Qingdao Agricultural University IACUC.

## Author Contributions

YD, XB, CX, and RL designed the study. RL and XL conducted the experiments, analyzed the data, and wrote the manuscript. RL, XL, and XB collected the data and performed the data analyses. All authors read and approved the final manuscript.

## Conflict of Interest

The authors declare that the research was conducted in the absence of any commercial or financial relationships that could be construed as a potential conflict of interest.

## Footnotes

1 https://pypi.org/project/goatools/

2 http://kobas.cbi.pku.edu.cn/

3 http://string-db.org/

## Abbreviations

CB1: cannabinoid receptor 1
PPAR γ 2: peroxisome proliferator activated receptor gamma
PLIN1: perilipin 1
HSL: hormone-sensitive lipase
Smad2: SMAD family member 2
Pi3K: phosphatidylinositol 3-kinase
IGF1R: insulin like growth factor 1 receptor
FASN: fatty acid synthase
GLUT1: glucose transporter 1
ACSL1: acyl-CoA synthetase long-chain family member 1
Insig1: insulin induced gene 1
C14:0: Myristic acid
C16:0: Palmitic acid
C18:0: Stearic acid
C17:0: Margaric acid
C16:1: Palmitoleic acid
C14:1: Myristoleic acid
C18:1: Oleic acid
C20:0: Arachidonic acid
C18:2n6c: Linoleic acid
C18:2n6t: Linolenic acid.
