# Supplementary material for: A Study of the Regulatory Mechanism of the CB1/PPARγ2/PLIN1/HSL Pathway for Fat Metabolism in Cattle
Source: Front Genet. 2021 May 4;12:631187. doi: 10.3389/fgene.2021.631187 (PMC8129027; doi:10.3389/fgene.2021.631187)
Supplement: Supplementary file 1 [file Data_Sheet_1.docx]

Title： **A study of the regulatory mechanism of the CB1/PPARγ2/HSL pathway for fat metabolism in cattle**

Author list：Ruili Liu^1^,^✝^, Xianxun Liu ^2^,^✝^, Xuejin Bai^1,2^, Chaozhu Xiao^1, 2^,Yajuan Dong^1, 2,*^

*Correspondence: [etcenter@126.com](mailto:etcenter@126.com)

^1^Laboratory of Animal Physiology and Biochemistry, Animal Embryo Center, College of Animal Science, Qingdao Agricultural University, Qingdao, Shandong, People’s Republic of China 200109.

^2^Laboratory of Animal Molecular Black Cattle Breeding Engineering Technology Center, College of Animal Science, Qingdao Agricultural University, Qingdao, Shandong, People’s Republic of China 200109.

^✝^ These authors contributed equally to this work.

Full list of author information is available at the end of the article.

List of E-mail:

Ruili Liu: [cream5423@126.com](mailto:hexingxing104@163.com); Xianxun Liu: xun[17854231458@126.com](mailto:281923989@qq.com);

Xuejin Bai: [blackcattle@126.com](mailto:985352431@qq.com); Chaozhu Xiao: [1245915136@qq.com](mailto:281923989@qq.com);

Yajuan Dong: [etcenter@126.com](mailto:ljf3070@126.com);

**Table S1** List of Significantly Enriched KEGG Pathways Related to Lipid Metabolism

| Pathways | ID | No. of DEGs | *P*-value | Upregulated genes | Downregulated genes |
| --- | --- | --- | --- | --- | --- |
| Regulation of lipolysis in adipocytes | Ko04923 | 13 | 0.00093 | *ADCY4, TAHR, PIK3R3, ADCY1, NPY1R* | *PTGS1, FABP4, PDE3B, ADORA1, ADCY7, LIPE, PLIN1, PTGER3* |
| Adipocytokine signaling pathway | Ko04920 | 11 | 0.039847 | *RXRA, SOCS3, NFKBIA, PPARGC1A, ACSBG2* | *PCK1, G6PC, LEPR, SLC2A1, LEP, ADIPOQ* |
| PPAR signaling | Ko03320 | 12 | 0.032492 | *PXPA, FADS2, ACSBG2* | *PLIN2, FABP7, PCK1, FABP4, FABP3, PLIN1, GK, ADIPOQ, SCD5* |
| ECM-receptor interaction | Ko04512 | 16 | 0.000853 | *COL6A1, COL6A2, LOC530102, COL4A2, ITGA6, LAMA5, COL1A1, HSPG2, DAG1, LAMB2, LAMA3, COL4A6, COL4A5, COL6A3, COL1A2, ITGB8* |  |
| AMPK signaling pathway | Ko04512 | 18 | 0.018498 | *PFKFB3, CREB3L1, PFKFB2, EEF2K, PIK3R3, PFKM, PPARGC1A* | *ADRA1A, FBP1, PCK1, LIPE, CAB39, G6PC, LEPR, LEP, ADIPOQ, SCD5, PPP2R2C* |
| Focal adhesion | Ko04510 | 24 | 0.017491 | *COL6A1, COL6A2, LOC530102, COL4A2, COL4A2, ITGA6, LAMA5, MYLK3, COL1A1, LAMB2, VEGFA, MYLK4, LAMA3, MYLPF, PAK1, COL4A6, COL4A5, PIK3R3, COL6A3, COL1A3, ITGB8, PDGFB, KDR* | *DIAPH1, MYL12A* |

**Table Specific primers-mouse**

| Gene | GeneBank accession number | Primer Sequence 5'→3' | Product Length/bp | Annealing temperature/℃ |
| --- | --- | --- | --- | --- |
| *CB1* | *U40709.1* | F:TTCAAGGAGAACGAGGAC  R:AGGGTGAGGGACAGGA | 113 | 50 |
| *PPARγ2* | *AF 156666.1* | F:TTATTGACCCAGAAAGCGAT  R:TATGGCACTTTGGTAGTCCTG | 290 | 56 |
| *PLIN1* | *NM_001308145.1* | F:CCTCCAAGGATGAGAACCA  R:TCAGGGCATCGGATAGGG | 306 | 56 |
| *HSL* | *U08188.1* | F:TTCTGTTCAAACTGGGTG  R:TGATGCTCTTCTGGGTC | 387 | 52 |

EXPOSURE OF THE ORIGINAL IMAGE：


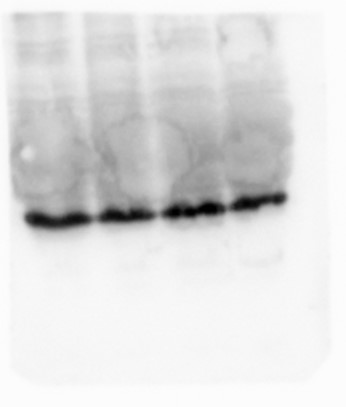

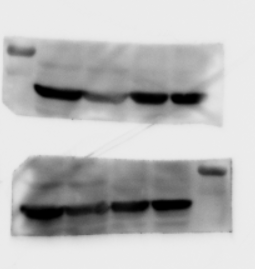


**GAPDH**

**Smad2**


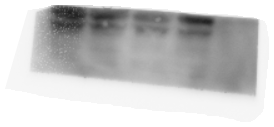

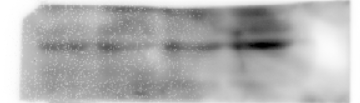


**Pi3k**


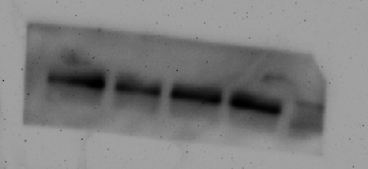


**GAPDH**


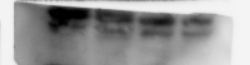

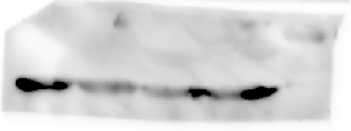


**TAK1**


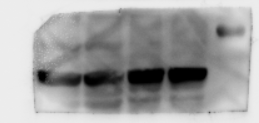


**GAPDH**

**
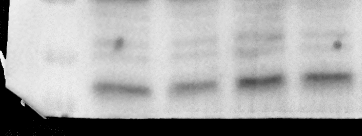
**
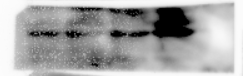

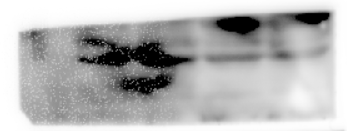


**IGF1R**


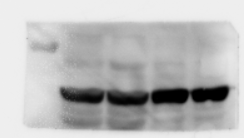


**（FIG.2）**

**GAPDH**


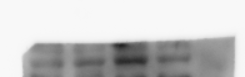

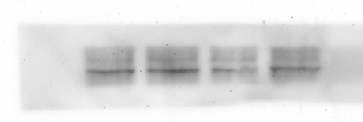


**FASN**


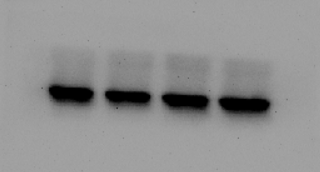


**（FIG.3）**

**GAPDH**

**
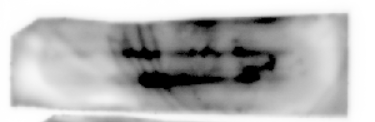
**
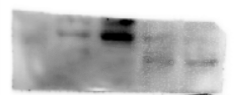

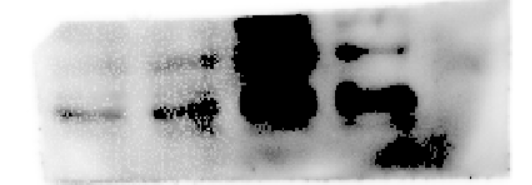


**ACSL1**

The results of the target protein test, the next band is the results of other target proteins, the results are not good, and were not used in the manuscript.

There was no test result of the target protein.


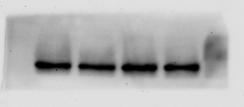


**GAPDH**


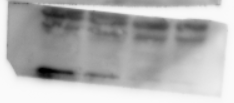

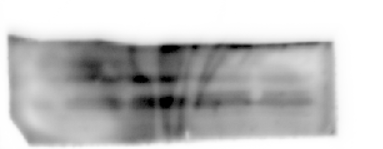


**GLUT1**


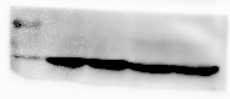


**GAPDH**


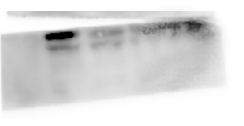

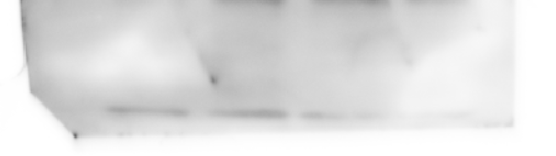

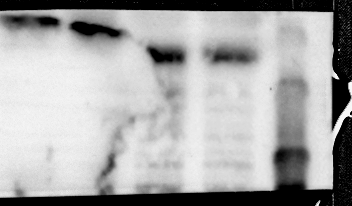


**Insig1**

Because the PVDF membrane was cut, a part of the target protein band was accidentally subtracted.

Repeated two groups of previous no results of the target protein test, the current present results of the previous group of experiments.


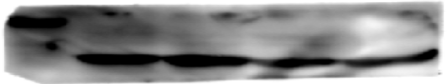


**NC-OE**

**NC-Ihitibor**

**CB1-OE**

**CB1-Ihitibor**

**GAPDH**


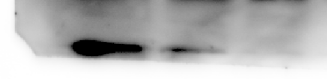

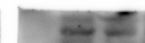

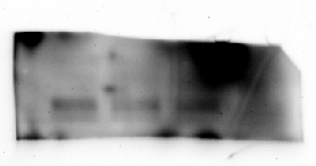


**CB1**


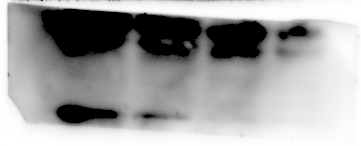

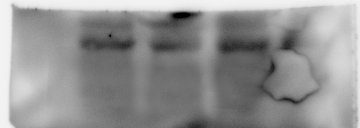


In the first experiment, the results of the latter two groups did not appear, but the former two groups had good effects.

In the second experiment, we repeated the two groups, but in the second group, the PVDF membrane was not washed clean, resulting in unclear bands.


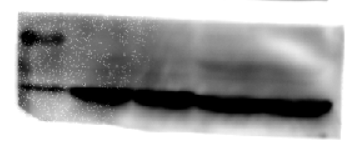


**GAPDH**

**NC-OE**

**NC-Ihitibor**

**CB1-OE**

**CB1-Ihitibor**


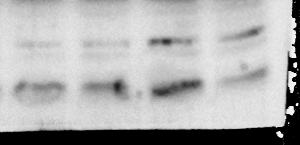

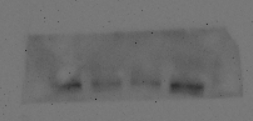


**PPARγ2**


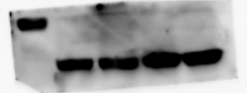


**GAPDH**

**CB1-OE**

**NC-OE**

**NC-Ihitibor**

**CB1-Ihitibor**


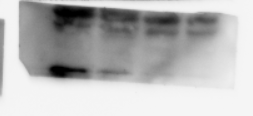
**
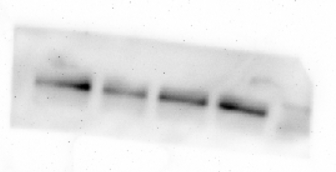
**

**PLIN**


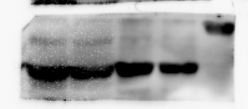


**GAPDH**


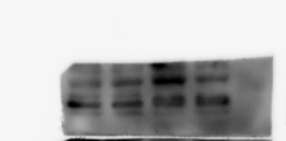

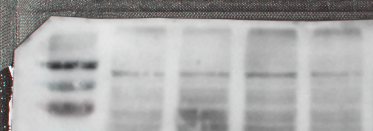


**HSL**

**NC-OE**

**NC-Ihitibor**

**CB1-OE**

**CB1-Ihitibor**


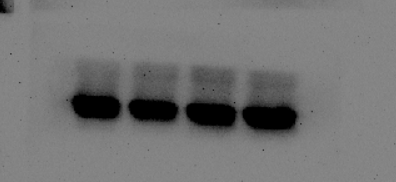


**（FIG.4）**

**GAPDH**


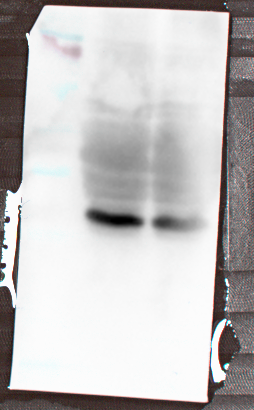


**FIG.6**


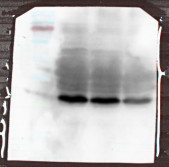

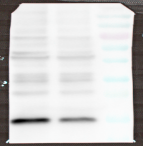

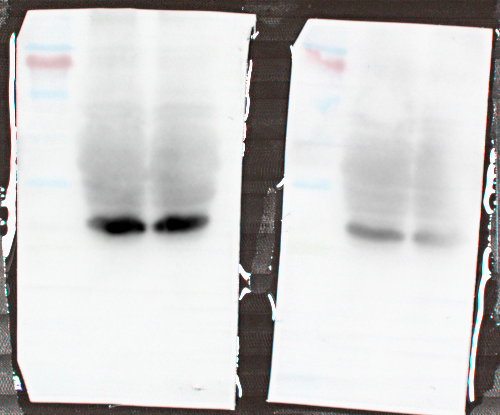

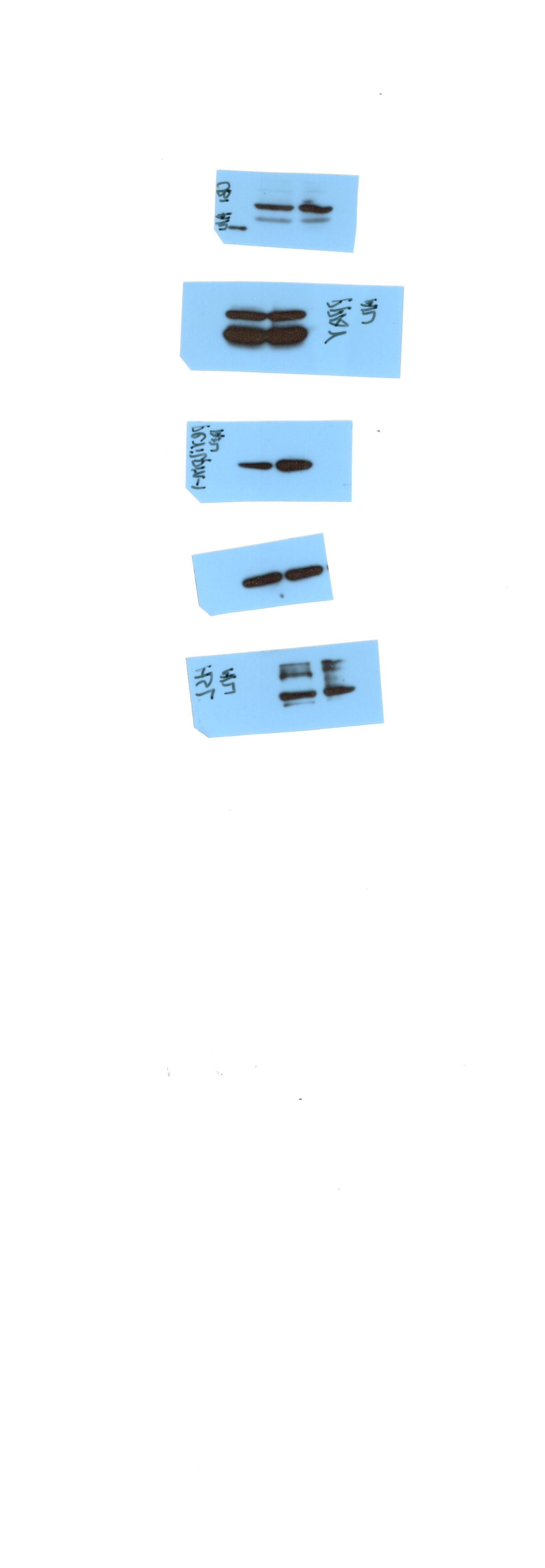


**HSL**

**GAPDH**

**PLIN1**

**PPARγ2**

**CB1**

NOTE：Because Western blot test crosscutting PVDF film, so we have provided the original picture for your identification, to ensure the authenticity of the test data.

Because of our limited cellular protein, we separately incubated the antibodies by cutting PVDF membrane. Finally, it shows the state of tailoring.

The specific test process is as follows:

Cell and tissue samples were lysed in RIPA buffer containing a protease inhibitor cocktail. Determination of protein concentration by BCA Kit (Beyotime Institute of Biotechnology, Shanghai, PR China).

(1)Electrophoresis: first 100 V constant voltage, after the sample enters the separation gel, adjust the voltage to 120 V constant pressure electrophoresis until the sample runs to the lowest layer. The gel was placed in the film buffer at room temperature for 30 min according to pre dyeing Marker gel.

(2) turn the membrane: from the positive pole to the negative electrode, the sponge filter paper - PVDF membrane - gel - filter paper - sponge, pay attention to removing bubbles, clamp the layers, put them into the electrobath, constant current 220 mA, 2h.

(3) PVDF membrane was cleaned with 1 × tbst solution for 3 times, 5 min each time.

(4) Add sealing solution, room temperature, 3 h.

(5) Tbst was washed 4 times, 5 min each time.

(6) The PVDF membrane was cut according to the pre staining maker to ensure that the internal reference protein and the target protein were respectively incubated with the first antibody. (this is a step leading to tailoring. At the same time, we cut PVDF membrane according to maker size to ensure that the target protein can be incubated separately. I believe that in the current experiment, many researchers adopt this method. One is to ensure the accuracy of the test results, and the other is to save the amount of protein used in the test.)

(7) One antibody was added and incubated overnight at 4 ℃.

(8) 1 × tbst solution was used to clean PVDF membrane for 4 times, 5 min each time.

(9) The Goat anti rabbit IgG (H + L) (1:2000) was labeled with the second anti horseradish peroxidation and incubated at room temperature for 1 h.

(10) The PVDF membrane was washed with 1 × tbst solution for 4 times, 5 min each time, and 3 times with distilled water for 5 min each time.

(11) Using the light-emitting kit, the negative film was exposed and developed in the dark room. ECL light-emitting substrate was exposed to develop the scanning film, and the optical density value of the target band was analyzed by Image J 1.39u.

Note: The pictures that I clearly guide us are not perfect, but it is true that the results of our experiments are presented in real terms. Due to the problem of our experimental operation, resulting in the result of the image clipping, is indeed our previous work negligence. In the future, we will improve the test method and try not to cut it. Finally, thank you for your patience as we have revised the manuscript many times.
